# A Highly Efficient Fluorescent Sensor Based on AIEgen for Detection of Nitrophenolic Explosives

**DOI:** 10.3390/molecules28010181

**Published:** 2022-12-25

**Authors:** Dongmi Li, Panpan Lv, Xiao-Wen Han, Zhilei Jia, Min Zheng, Hai-Tao Feng

**Affiliations:** 1 Henan Key Laboratory of Function-Oriented Porous Materials, College of Chemistry and Chemical Engineering, Luoyang Normal University, Luoyang 471000, China; 2 AIE Research Center, College of Chemistry and Chemical Engineering, Baoji University of Arts and Sciences, Baoji 721013, China

**Keywords:** fluorescence probe, aggregation-induced emission, trinitrophenol, explosives

## Abstract

The detection of nitrophenolic explosives is important in counterterrorism and environmental protection, but it is still a challenge to identify the nitroaromatic compounds among those with a similar structure. Herein, a simple tetraphenylethene (TPE) derivative with aggregation-induced emission (AIE) characteristics was synthesized and used as a fluorescent sensor for the detection of nitrophenolic explosives (2, 4, 6-trinitrophenol, TNP and 2, 4-dinitrophenol, DNP) in water solution and in a solid state with a high selectivity. Meanwhile, it was found that only hydroxyl containing nitrophenolic explosives caused obvious fluorescence quenching. The sensing mechanism was investigated by using fluorescence titration and ^1^H NMR spectra. This simple AIE-active probe can potentially be applied to the construction of portable detection devices for explosives.

## 1. Introduction

The effective detection of nitroaromatic compounds (NACs) is of great importance in modern society owing to their threats to both national security and environmental safety. Different nitroaromatic explosives such as 2, 4, 6-Trinitrophenol (TNP, picric acid) and 2, 4-dinitrophenol (DNP) are broadly utilized in the assembling of rocket fills, firecrackers, matches, etc. Additionally, TNP and its derivatives are also used in the field of medical formulation as an antiseptic agent, as well as being used as a yellow pigment in dye and leather industries, which will cause environmental pollution and health hazards [1,2]. Consequently, the development of highly selective and sensitive sensors with a quick response for discriminating TNP and DNP is imperative for natural remediation, regular citizen security, and military tasks.

Presently, several analytical techniques have been developed for the detection of nitroaromatic explosives, such as trained canines [3], mass spectrometry [4], gas chromatography [5,6], liquid chromatography [7], ion mobility spectrometry [8], electrochemical assay [9], high performance liquid chromatography [10], X-ray imaging, Raman spectroscopy [11], and so on. In contrast to the above-mentioned methods, fluorescent probes with the merits of high sensitivity, selectivity, easy visualization, and a short response time have attracted great attention in various areas [12,13,14,15,16]. To date, numerous fluorescent sensors based on polymers [17,18], metal–organic frameworks [19], fluorescent quantum dots [20,21], and organic small molecules [13] have been designed and developed. Despite these advances, most of the traditional fluorophores have often encountered the aggregation-caused quenching (ACQ) problem in the aggregated state, which has been a hurdle to fluorescence sensing. Moreover, selective detection of nitroaromatic explosives remains difficult due to their similar electron affinity.

Since aggregation-induced emission (AIE) was termed by Tang in 2001 [22], a large number of AIE luminogens (AIEgens) have been developed for photoelectric devices, biomaterials and fluorescent probes [23,24,25,26,27]. Thanks to their high sensitivity and solid-state emission efficiency, detection of explosives based on AIE-active probes has received a lot of interest [28,29,30,31,32]. Among these AIEgens, tetraphenylethene (TPE) is the most popular building block for the construction of fluorescence sensors due to its high photostability, simple synthesis, and easy structure modification. For example, Tang and their co-workers reported that a series of TPE-based polymers can serve as highly sensitive sensors for TNP through a mode of emission quenching with this substrate [33,34,35,36]. To provide the fluorescence sensors with better selectivity, the Zheng group designed several TPE-based macrocycles showing a high selectivity for NACs, alongside the excellent sensitivity by virtue of the encapsulation [37,38,39]. Throughout the course of our continuous effort to develop an excellent fluorescence probe for explosives, herein a simple TPE-based AIEgen was synthesized to detect TNP from a series of NACs. Such material showed highly sensitive fluorescence quenching to TNP in aqueous solution and in a solid state by virtue of photo-induced electron transfer. This simple AIE-active probe can potentially be applied to the construction of portable detection devices for explosives.

## 2. Results and Discussion

**Scheme 1:**
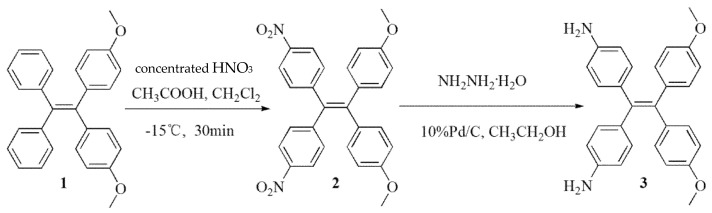
Synthetic route of compound **3**.

The synthetic procedure of the target compound **3** was designed and presented in Scheme 1. The known tetraphenylethene derivative **1** was utilized as the starting material. It was then nitrated by concentrated nitric acid and acetic acid in dichloromethane to afford molecule **2**. By a reduction of **2** with hydrazine hydrate and Pd/C in ethanol, the target molecule **3** was obtained in a good yield of 87%. The molecule **3** was fully characterized by ^1^H NMR, ^13^C NMR, high resolution mass spectra (HRMS), and infrared (IR) spectra in the Supporting Information (Figures S1–S4).

**Figure 1:**
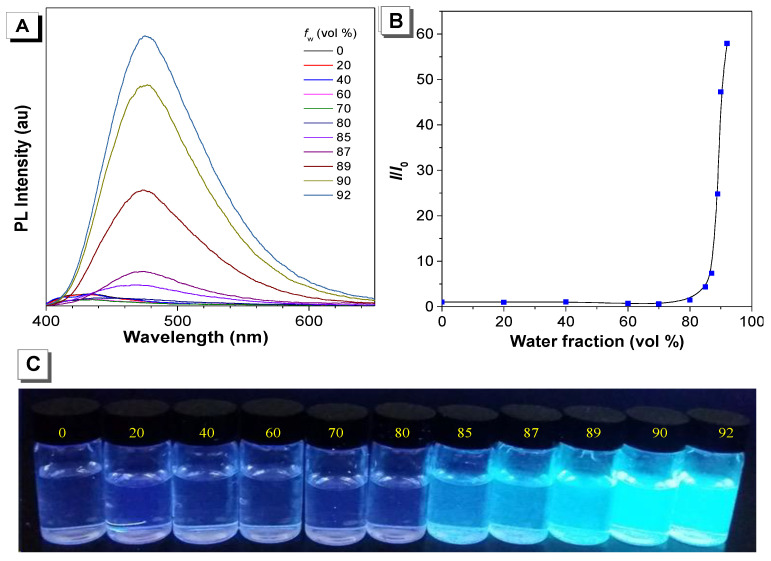
(**A**) Fluorescence spectra of compound **3** (5.0 × 10^−5^ M) in THF with different water fractions. λ_ex_ = 340 nm, ex/em slit widths = 5/5 nm. (**B**) PL intensity ratio of *I*/*I*_0_ in different THF/H_2_O mixtures. (**C**) Fluorescent images of compound **3** (5.0 × 10^−5^ M) with changing water fractions from 0 to 92% in THF under a 365 nm UV lamp.

The photophysical properties of compound **3** were then investigated. The UV/Vis absorption spectra of **3** responding to TNP were measured in dimethylsulfoxide (DMSO). The molecule **3** showed absorption bands at 342, 292 and 261 nm. When adding TNP to **3**, the absorption intensity largely increased, but the algebraic sum of spectrum of **3** and the spectrum of TNP is similar to the spectrum of the mixture of **3** + TNP, implying that there were weak interactions in the solution state (Figure S5). From the photoluminescence (PL) spectra we can see that compound **3** was non-emissive in THF (Figure 1). Upon the addition of poor solvent water to THF, the solution remained weak in fluorescence from 0 to 80% water fraction. Upon further enhancing the water fraction to 92%, compound **3** emitted bright blue fluorescence at 478 nm. The 58-fold enhancement of PL intensity indicated that compound **3** is an AIE-active compound. The scanning electron microscope (SEM) images revealed that the turbid solution of **3** in 92% water fraction was composed of many rod-like aggregates with the length of micrometers (Figure S6).

**Figure 2:**
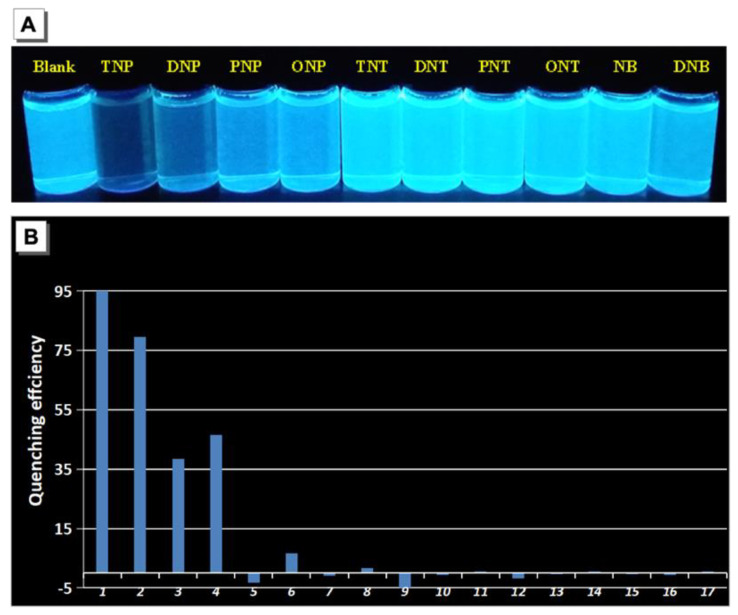
(**A**) Fluorescent images of compound **3** with various nitrophenolic explosives under a 365 nm UV lamp. (**B**) The Fluorescence quenching efficiencies ((1 − *I*/*I*_0_) × 100%), where I and I_0_ denote the fluorescence intensity of compound **3** with and without analytes, respectively. 1: TNP, 2: DNP, 3: PNP, 4: ONP, 5: TNT, 6: DNT, 7: PNT, 8: ONT, 9: NB, 10: DNB, 11: DNFB, 12: HPN, 13: DNCB, 14: 3−Cyanopheno, 15: Phenol, 16: DNA, 17: HBN. Solvent: H_2_O: THF = 9: 1, [**3**] = [explosives]/2 = 2 × 10^−5^ M. λ_ex_ = 340 nm, ex/em slit widths = 5/5 nm.

The fluorescence response of molecule **3** to NACs was then studied in H_2_O/THF (9: 1, *v*/*v*). As shown in Figure 2A, molecule **3** (20 μM) fluoresced strongly in 90% water fraction without NACs. When different nitrophenolic explosives (40 μM) were added into the solution, such as 2,4,6-trinitrophenol (TNP), 2,4-dinitrophenol (DNP), p-nitrophenol (PNP), o-nitrophenol (ONP), 2,4,6-trinitromethylbenzene (TNT), 2,4-dinitrotoluene (DNT), p-nitrotoluene (PNT), o-nitrotoluene (ONT), nitrobenzene (NB), 1,3-dinitrobenzene (DNB), 1-fluoro-2,4-dinitrobenzene (DNFB), 4-hydroxyisophthalonitrile (HPN), 2,4-dinitrochlorobenzene (DNCB), 3-cyanopheno (CP), phenol, 3,5-dinitrobenzoic acid (DNA), and 2-Hydroxybenzonitrile (HBN), the sharp quenching caused by TNP and DNP was observed under a 365 nm UV light. Meanwhile, it was found that only hydroxyl containing nitrophenolic explosives caused obvious fluorescence quenching, while other NACs showed a minor influence on the emission of molecule **3**. The quenching efficiency of PNP, ONP, DNP, and TNP increased with the enhancement of acidity. Therefore, it is inferred that the existence of electrostatic interactions led to the high selectivity of molecule **3** to TNP and DNP. The quenching efficiency ((1 − *I*/*I*_0_) × 100%) of compound **3** for TNP and DNP were 95% and 79%, respectively (Figure 2B and Figure S7).

**Figure 3:**
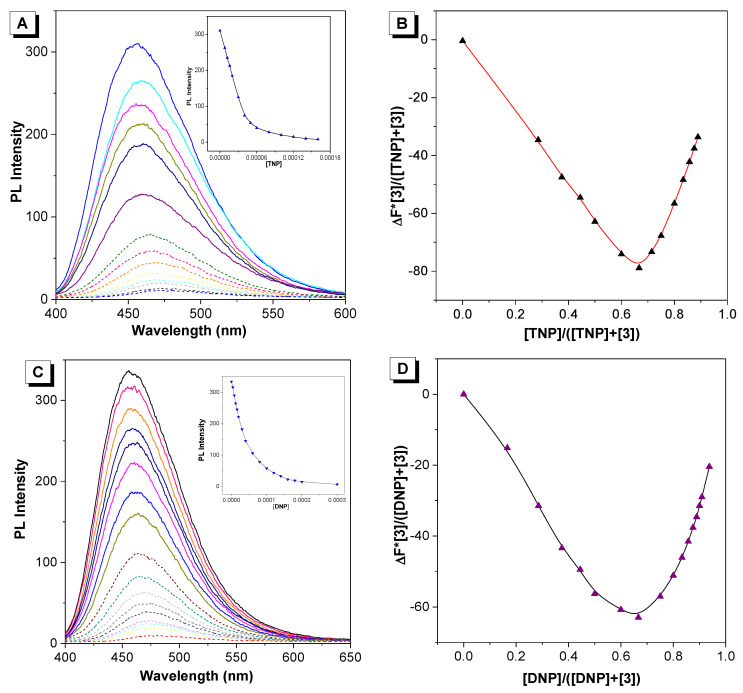
(**A**) The PL spectra of compound **3** with different amounts of TNP. Solvent: H_2_O: THF = 9: 1, [**3**] = 2 × 10−5 M, [TNP] = 0–1.6 × 10−4 M. Inset: the PL change was observed at 460 nm when compound 3 was combined with TNP. (**B**) The Job’s plot of compound 3 (2 × 10−5 M) with TNP at 460 nm. (**C**) The PL spectra of compound 3 with different amounts of DNP. Solvent: H2O: THF = 9: 1, [**3**] = 2 × 10−5 M, [DNP] = 0–3 ×1 0−4 M. Inset: compound 3 fluorescence intensity variation with TNP content at 460 nm. (**D**) The Job’s plot of compound 3 (2 × 10−5 M) with DNP at 460 nm. λex = 340 nm, ex/em slit widths = 5/5 nm.

The fluorescence titration of **3** (20 μM) with different equivalents of TNP was measured to verify their complexation ratio. As shown in Figure 3A, the PL intensity of molecule **3** at 460 nm gradually decreased when the TNP concentration was increased to 40 μM. The stoichiometry between compound **3** and TNP was calculated from Job’s plot to be 1: 2 (Figure 3B). Based on the data presented in Figure S8, the association constant was estimated to be 3.4 × 10^8^ from linear curve fitting using Origin software [40,41]. Similar results were obtained from the fluorescence titrations of **3** with DNP (Figure 3C). The stoichiometry from Job’s plot was estimated to be 1:2 also (Figure 3D) and the association constant was calculated to be 7.4 × 10^7^ (Figure S9). From the association constants we can conclude that molecule **3** has much a stronger affinity to TNP than DNP. Due to the higher number of electron-withdrawing nitro groups in TNP, it exhibited much more acidity than other nitrophenolic compounds. Thus, it is easy to form complexes with molecule **3** to occur photo-induced electron transfer within the complex resulting in fluorescence quenching. Then fluorescence lifetimes of compound **3**, TNP, and the mixture of compound **3** + TNP were measured in an aqueous solution to verify their interactions. As illustrated in Figure S10, compound **3** gave a lifetime of 4.52 ns. Upon the addition of TNP to the system, the lifetime reduced to 0.35 ns, indicating the existence of static fluorescence quenching.

**Figure 4:**
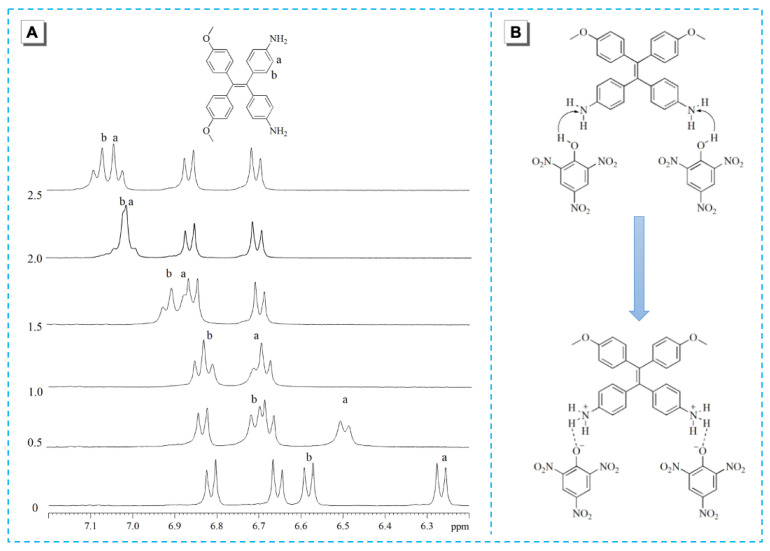
(**A**) Changes of ^1^H NMR spectra of compound **3** (5 mM) in *d*_6_-DMSO with the addition of TNP. (**B**) The possible binding mode of compound **3** with TNP.

^1^H NMR titration in *d*_6_-DMSO was performed again to gain a deeper understanding of the bond formation between compound **3** and TNP. In fact, we made an attempt to record the NMR titration in the mixture of deuterated water and THF with 90% deuterated water fraction. Unfortunately, the compound **3** and TNP could not dissolve in the mixed solvents. Thus, we carried out ^1^H NMR titration in *d*_6_-DMSO. As shown in Figure 4A, when two equivalents of TNP were added, the chemical shift H_a_ of compound **3** showed a downfield shift from 6.26 to 7.01 ppm. Upon further addition of TNP, the chemical shift was only slightly altered. Additionally, there was also an obvious downfield shift of H_b_ in compound **3** from 6.58 to 7.02 ppm. These results proved that the electrostatic interaction between amino groups and hydroxyl group mainly contributes to the binding between TNP and compound **3**. ^1^H NMR titration indicated that a 1:2 binding ratio was obtained between compound **3** and TNP as shown in Figure S11. It is worth noting that this binding ratio was obtained from a *d*_6_-DMSO concentrated solution (5 mM) in ^1^H NMR titration and it is not clear if this will also be true with its diluted solution in H_2_O: THF (90% water fraction), with concentration of the order 10^−5^ M. However, from the results of ^1^H NMR and fluorescence titration we can see that they obtained the same complexation ratio. Additionally, Figure 4B illustrates a possible binding mode between compound **3** and TNP.

**Figure 5:**
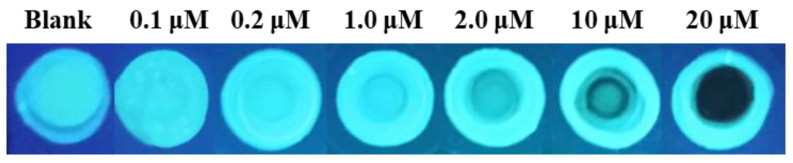
Fluorescent photographs of compound **3** with and without TNP solution on the TLC plate under 365 nm UV light.

Furthermore, fluorescence detection of TNP was also estimated in the solid state (Figure 5). Compound **3** was dripped onto the TLC plate and dried under a vacuum. The TLC plate with pure compound **3** showed bright fluorescence. When different concentrations of TNP solution were dripped on the spot of compound **3**, the fluorescence was quickly quenched. From the picture we can see that 0.2 μM of compound **3** can cause obvious fluorescence quenching on the TLC plate. The data verified that compound **3** is an excellent sensor for TNP not only in the solution, but also in the solid state.

## 3. Materials and Methods

Materials: All reagents and solvents were chemical pure (CP) grade or analytical reagent (AR) grade and were used as received.

Measurements: ^1^H and ^13^C NMR were measured on 400 MHz Bruker Advanced III (Bremen, Germany). Mass spectrum was measured on Waters instrument (Framingham, MA, USA). IR was measured on Bruker VERTEX70 (Bremen, Germany). Fluorescent spectra were collected on Hitachi F-4500 spectrophotometer (Tokyo, Japan).

### 3.1. Synthesis of ***2***

The mixture of 30 mL dichloromethane, 2.85 mL (40.9 mmol) concentrated nitric acid, and 2.34 mL (20.8 mmol) acetic acid was added to a 100 mL drying flask. The flask was placed in a low temperature reaction bath at –15 °C, 2 g 4, 4’-dimethoxytetraphenylethene (5 mmol) was added to the mixture under stirring for 0.5 h. The reaction mixture was taken out of the low-temperature reaction bath and returned to room temperature. The product was quenched by adding 20 mL of water. The organic phase was separated and washed with water several times until the pH was 7. Compound **2** was obtained as a yellow solid by evaporating the solvent using a rotary evaporator (1.95 g, 81%).

### 3.2. Synthesis of ***3***

Compound **2** (1 g, 2.1 mmol), 30 mL of absolute ethanol, hydrazine hydrate (1 mL, 21 mmol), and Pd/C (87 mg, 0.42), respectively, were added to the flask. After two hours of refluxing, the mixture was cooled to room temperature. The organic phase was obtained after filtration and then concentrated with a rotary evaporator. An ethyl acetate/petroleum ether 1/1 eluent was used to purify the crude product with a silica gel column. Compound **3** was obtained as a yellow powder with an 87% yield (0.77 g). Mp 216.4–218.6 °C; IR (KBr) *ν* 3460, 3414, 3363, 3024, 2835, 1605, 1508, 1443, 1238, 1172, 1026, 833 cm^−1^; ^1^H NMR (400 MHz, DMSO) δ 6.46 (d, *J* = 8.4 Hz, 4H), 6.63 (d, *J* = 8.4 Hz, 4H), 6.56 (d, *J* = 8.0 Hz, 4H), 6.24 (d, *J* = 8.0 Hz, 4H), 4.92 (s, 4H), 3.64 (s, 6H) ppm; ^13^CNMR (100 MHz, DMSO) δ: 156.9, 146.7, 139.9, 137.4, 134.6, 132.0, 131.7, 131.6, 113.1, 113.0 ppm; MS m/z calcd for C_28_H_26_N_2_O_2_ 422.5 [M], found 423.2055 [M^+^].

## 4. Conclusions

In conclusion, this paper presented the design and synthesis of an aggregation-induced emission fluorescent sensor for a nitrophenolic explosives in aqueous media, which was highly effective. The fluorescence quenching of the sensor was observed due to the formation of the 1: 2 complex with TNP/DNP. The binding constants of compound **3** with TNP and DNP were calculated to be 3.4 × 10^8^ and 7.4 × 10^7^, respectively. This simple sensor exhibited high potential for the detection of TNP and DNP both in water and in the solid state.

## Data Availability

Not applicable.

## Supplementary Materials

The following supporting information can be downloaded at: https://www.mdpi.com/article/10.3390/molecules28010181/s1, Figures S1–S4: characteristic spectra; Figure S5: UV/vis spectra; Figure S6: SEM images; Figures S7–S11: fluorescence spectra.

## Sample Availability

Samples of the compounds are not available from the authors.
