# Supplementary material for: A Highly Efficient Fluorescent Sensor Based on AIEgen for Detection of Nitrophenolic Explosives
Source: Molecules. 2022 Dec 25;28(1):181. doi: 10.3390/molecules28010181 (PMC9821835; doi:10.3390/molecules28010181)
Supplement: Supplementary file 1 [file molecules-28-00181-s001.zip › molecules-2071214-supplementary.pdf]

## Supporting Information

# A Highly Efficient Fluorescent Sensor Based on AIEgen for Detection of Nitrophenolic Explosives

**Dongmi Li [1,*,†], Panpan Lv [2,†], Xiao-Wen Han[2], Zhilei Jia [1], Min Zheng [1] and Hai-Tao Feng [2,*]**

[1]Henan Key Laboratory of Function-Oriented Porous Materials, and College of Chemistry and Chemical Engineering, Luoyang Normal University, Luoyang, Henan 471000, China

[2]AIE Research Center, College of Chemistry and Chemical Engineering, Baoji University of Arts and Sciences, Baoji, Shaanxi 721013, China

*Correspondence: lidongmi223@126.com (D. L.), haitaofeng907@163.com (H.-T. F.)

† These authors contributed equally to this work.

## Materials and Methods

**Materials**. All reagents and solvents were chemical pure (CP) grade or analytical reagent (AR) grade and were used as received.

### Measurements

$^{1}H$ and $^{13}C$ NMR were measured on 400 MHz Bruker Advanced III. Mass spectrum was measured on Waters instrument. IR was measured on Bruker VERTEX70. Fluorescent spectra were collected on Hitachi F-4500 spectrophotometer.

### Synthesis of 2

The mixture of 30 mL dichloromethane, 2.85 mL (40.9 mmol) concentrated nitric acid and 2.34 mL (20.8 mmol) acetic acid was added to a 100 mL drying flask. The flask was placed low temperature reaction bath at –15 °C, 2 g 4, 4'-dimethoxytetraphenylethene (5 mmol) was added to the mixture under stirring for 0.5 h. Take the reaction mixture out of the low-temperature reaction bath and return it to room temperature. The product was quenched by adding 20 mL of water. The organic phase was separated and washed with water several times until the pH is 7. Compound **2** was obtained as yellow solid by evaporating the solvent using a rotary evaporator (1.95 g, 81 %).

### Synthesis of 3

To the flask were added compound **2** (1 g, 2.1 mmol), 30 mL absolute ethanol, hydrazine hydrate (1 mL, 21 mmol), and Pd/C (87 mg, 0.42) in order. After two hours of refluxing, the mixture was cooled to room

temperature. The organic phase was obtained after filtration and then concentrated with a rotary evaporator. An ethyl acetate/petroleum ether 1/1 eluent was used to purify the crude product with a silica gel column. Compound **3** was obtained as yellow powder with an 87% yield (0.77 g). Mp 216.4–218.6 °C; IR (KBr) $\nu$ 3460, 3414, 3363, 3024, 2835, 1605, 1508, 1443, 1238, 1172, 1026, 833 cm$^{-1}$; $^{1}$H NMR (400 MHz, DMSO) $\delta$ 6.46 (d, $J$ = 8.4 Hz , 4H), 6.63 (d, $J$ = 8.4 Hz , 4H), 6.56 (d, $J$ = 8.0 Hz , 4H), 6.24 (d, $J$ = 8.0 Hz , 4H), 4.92 (s, 4H), 3.64 (s, 6H) ppm; $^{13}$CNMR (100 MHz, DMSO) $\delta$: 156.9, 146.7, 139.9, 137.4, 134.6, 132.0, 131.7, 131.6, 113.1, 113.0 ppm; MS m/z calcd for $C_{28}H_{26}N_2O_2$ 422.5 [M], found 423.2055 [M$^{+}$].

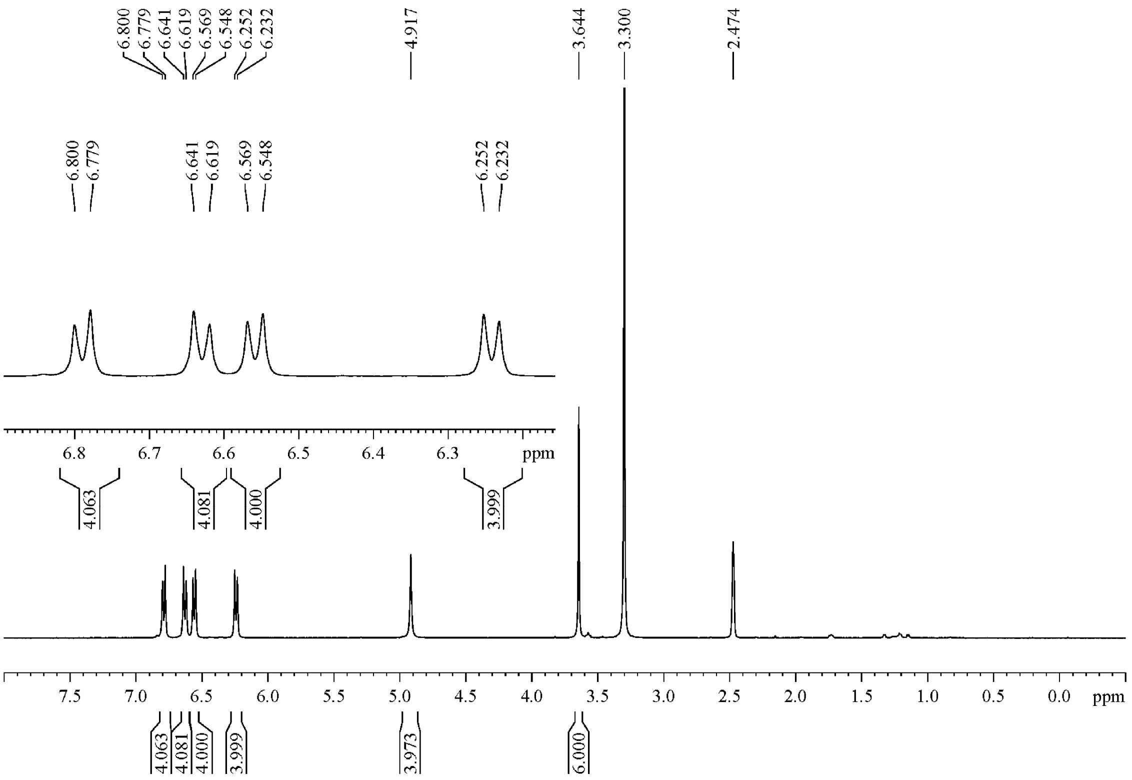


**Figure S1**. $^{1}$H NMR spectrum of compound **3** in DMSO.

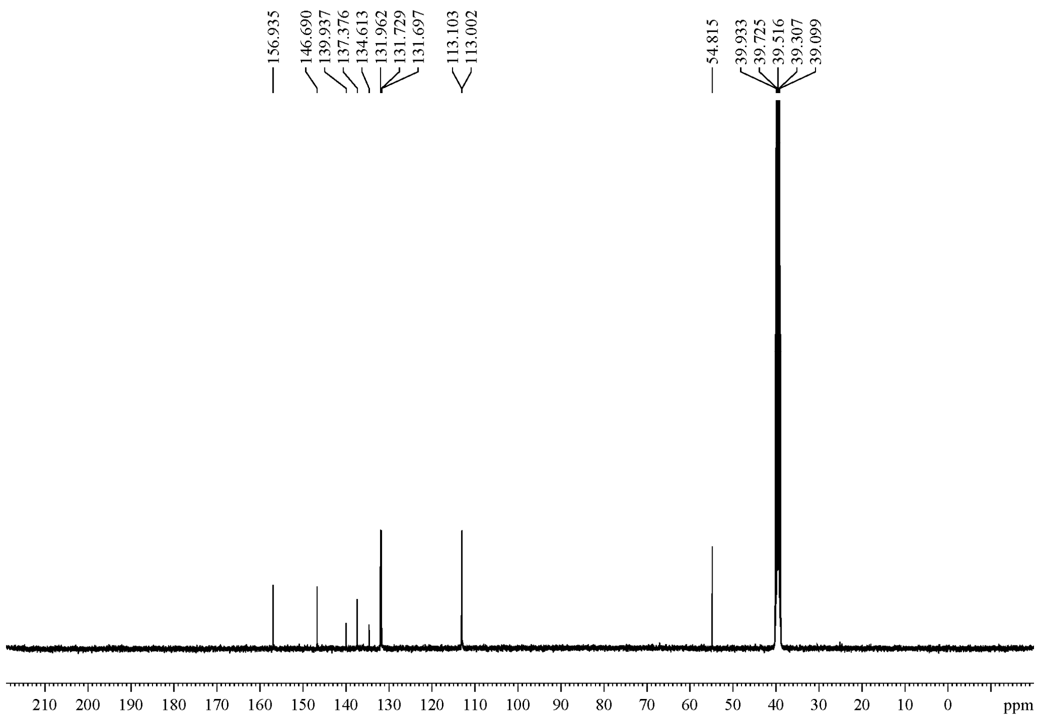


**Figure S2**. $^{13}C$ NMR spectrum of compound **3** in DMSO.

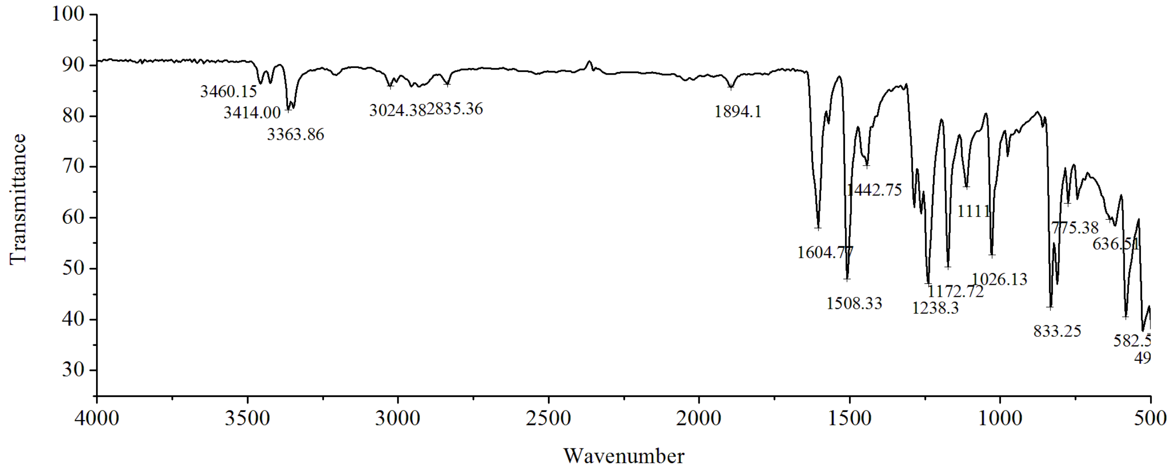


**Figure S3**. IR spectrum of compound **3**.

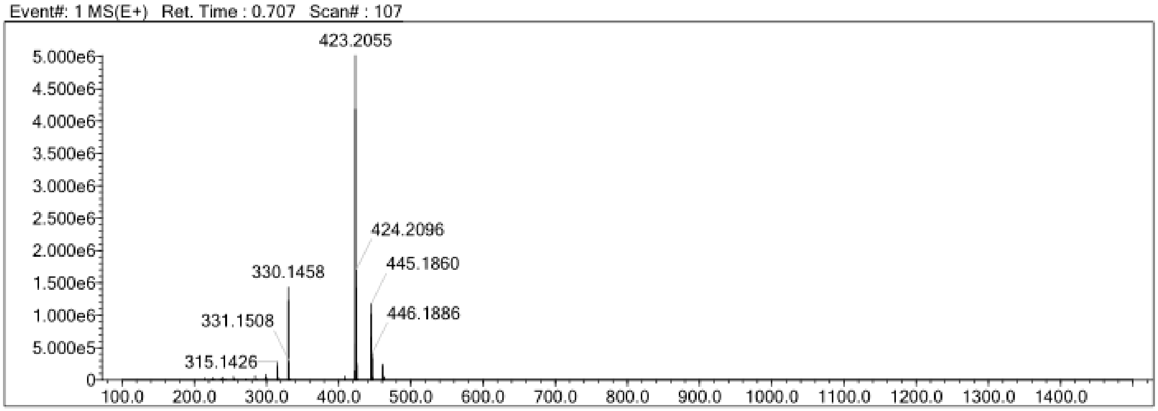


**Figure S4**. HR-MS spectrum of compound **3**.

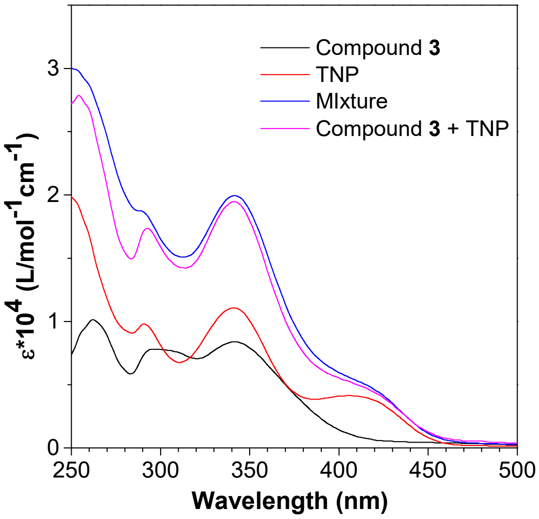


**Figure S5**. UV-vis spectra of compound **3**, TNP and compound **3**+TNP in DMSO, [**3**] = [TNP]/2 = 2×10$^{-5}$ M.

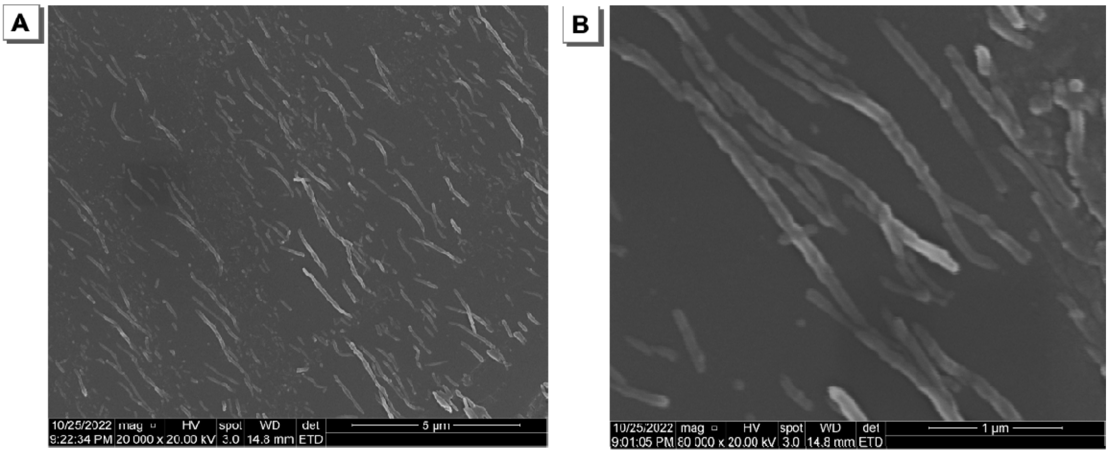


**Figure S6**. SEM images of the compound 3 in THF/$H_2O$ (V/V, 5/95).

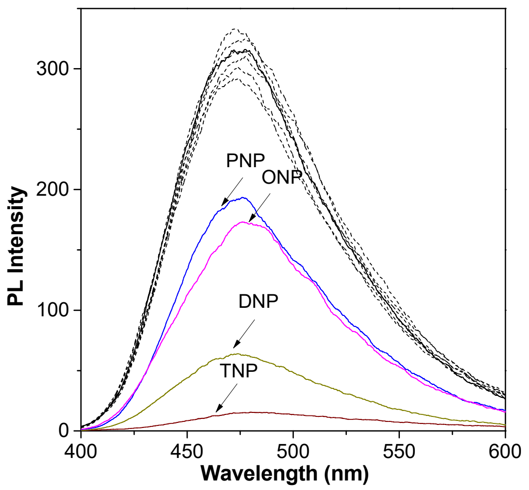


**Figure S7.** Fluorescence spectra of compound **3** in the presence of different explosives, solvent: $H_2O$: THF = 9: 1, [**3**] = [explosives]/2 = $2\times10^{-5}$ M, $\lambda_{ex}$ = 340 nm, ex/em slit widths = 5/5 nm.

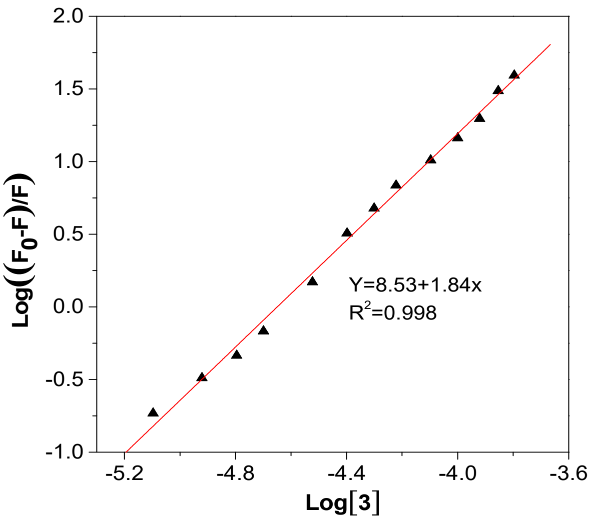


**Figure S8.** The plot of Log(($F_0$-F)/F) vs. Log[TNP] at 460 nm, solvent: $H_2O$: THF = 9: 1, [**3**] = $2\times10^{-5}$ M; [TNP] = 0–$16\times10^{-5}$ M.

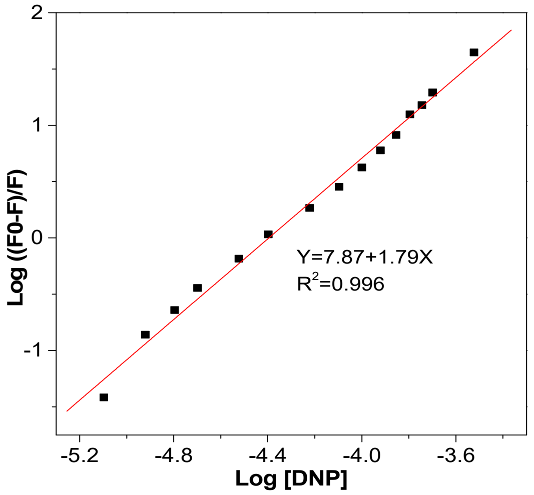


**Figure S9.** The plot of Log (($F_0$-F)/F) vs. Log [TNP] at 460 nm, solvent: $H_2O$: THF = 9: 1, [**3**] = $2\times10^{-5}$ M; [TNP] = 0–$16\times10^{-5}$ M.

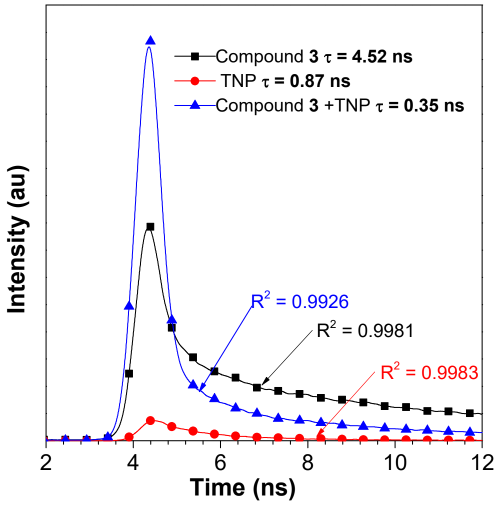


**Figure S10.** Fluorescence decay profiles of compound **3**, TNP and the mixture of compound **3** + TNP at 450 nm, $\lambda_{ex}$ = 340 nm.

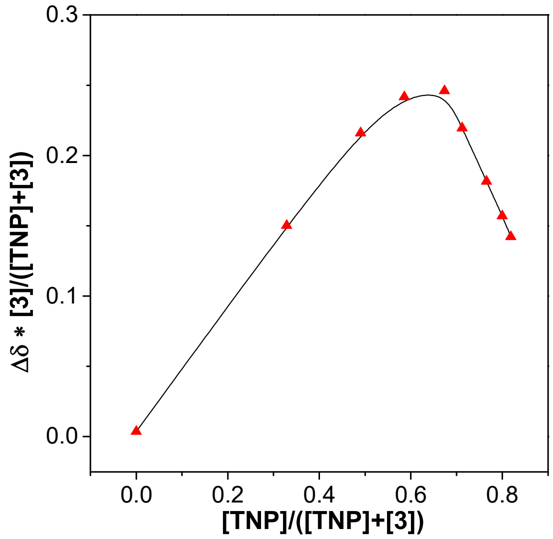


**Figure S11.** The Job plot for $^1$H NMR titration of compound **3** with TNP, solvent: DMSO-$d_6$; [**3**] = $5\times10^{-3}$ M; [TNP] = 0–$25\times10^{-3}$ M.
